# Structure of human promyeloperoxidase (proMPO) and the role of the propeptide in processing and maturation

**DOI:** 10.1074/jbc.M117.775031

**Published:** 2017-03-27

**Authors:** Irina Grishkovskaya, Martina Paumann-Page, Rupert Tscheliessnig, Johanna Stampler, Stefan Hofbauer, Monika Soudi, Benjamin Sevcnikar, Chris Oostenbrink, Paul G. Furtmüller, Kristina Djinović-Carugo, William M. Nauseef, Christian Obinger

**Affiliations:** From the ‡ Department of Structural and Computational Biology, Max F. Perutz Laboratories, University of Vienna, A-1030 Vienna, Austria,; the § Department of Chemistry, Division of Biochemistry, BOKU-University of Natural Resources and Life Sciences, Muthgasse 18, A-1190 Vienna, Austria,; the ¶ Austrian Centre of Industrial Biotechnology (ACIB), Muthgasse 11, A-1190 Vienna, Austria,; the ‖ Department of Material Sciences and Process Engineering, Institute of Molecular Modeling and Simulation, BOKU-University of Natural Resources and Life Sciences, A-1190 Vienna, Austria,; the ‡‡ Inflammation Program and Department of Medicine, Roy J. and Lucille A. Carver College of Medicine, University of Iowa, Iowa City, Iowa 52242, and; the ** Department of Biochemistry, Faculty of Chemistry and Chemical Technology, University of Ljubljana, Večna pot 113, 1000 Ljubljana, Slovenia

**Keywords:** biosynthesis, crystallography, heme, innate immunity, myeloperoxidase

## Abstract

Myeloperoxidase (MPO) is synthesized by neutrophil and monocyte precursor cells and contributes to host defense by mediating microbial killing. Although several steps in MPO biosynthesis and processing have been elucidated, many questions remained, such as the structure-function relationship of monomeric unprocessed proMPO *versus* the mature dimeric MPO and the functional role of the propeptide. Here we have presented the first and high resolution (at 1.25 Å) crystal structure of proMPO and its solution structure obtained by small-angle X-ray scattering. Promyeloperoxidase hosts five occupied glycosylation sites and six intrachain cystine bridges with Cys-158 of the very flexible N-terminal propeptide being covalently linked to Cys-319 and thereby hindering homodimerization. Furthermore, the structure revealed (i) the binding site of proMPO-processing proconvertase, (ii) the structural motif for subsequent cleavage to the heavy and light chains of mature MPO protomers, and (iii) three covalent bonds between heme and the protein. Studies of the mutants C158A, C319A, and C158A/C319A demonstrated significant differences from the wild-type protein, including diminished enzymatic activity and prevention of export to the Golgi due to prolonged association with the chaperone calnexin. These structural and functional findings provide novel insights into MPO biosynthesis and processing.

## Introduction

Neutrophils figure prominently in human host defense against infection, and optimal antimicrobial action in neutrophils relies on the action of hypochlorous acid (HOCl), the product of the myeloperoxidase (MPO)^4^-H_2_O_2_-chloride system (1). Production and targeting of MPO to azurophilic granules occur in promyelocytic myeloid precursors in normal human bone marrow (2), and only neutrophils and monocytes express MPO. However, MPO shares many structural features with other members of the peroxidase-cyclooxygenase superfamily (3, 4), most notably the presence of covalent bonds between the heme group and the peptide backbone (5, 6). In MPO, the prosthetic group is covalently linked to the protein via autocatalytic formation of two ester bonds with highly conserved aspartate and glutamate residues and a sulfonium ion linkage between the 2-vinyl group and a conserved methionine (5, 7–9). The existence of these three covalent heme-to-protein bonds correlates with the peculiar spectroscopic, redox, and catalytic properties of this metalloprotein (6, 10–12) and its capacity to catalyze hypochlorous acid production in phagosomes of stimulated human neutrophils (13).

Among members of the peroxidase-cyclooxygenase superfamily, mature MPO alone is a functional dimer, composed of two identical glycosylated protomers that contain covalently bound heme, an N-terminal 14.5-kDa light polypeptide (L-chain), and a 59-kDa heavy polypeptide (H-chain) (5, 7, 8), with the two H-chains linked covalently by a single Cys-319–Cys-319 bridge. Each heavy-/light-chain subunit-containing protomer (*i.e.* hemi-MPO) exhibits the same specific peroxidase activity as does the holoenzyme (14), suggesting that dimerization contributes little to the overall enzymatic activity of mature MPO and leaving the structural or functional advantages of dimerization unknown.

**Figure 1.**
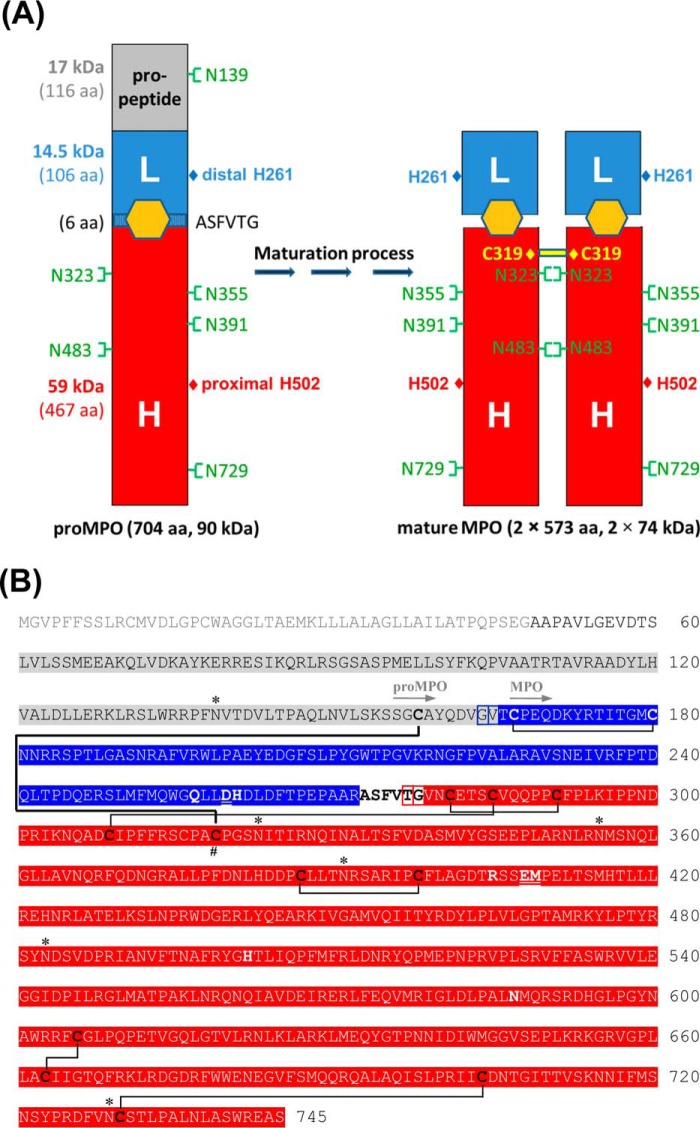
**Structure and sequence of proMPO and mature MPO.** *A*, schematic presentation of the structure of monomeric unprocessed proMPO and dimeric mature MPO, including the locations of the distal and proximal catalytic histidines (His-261 and His-502) and the *N*-glycosylation sites. The propeptide of proMPO and the L- and H-chain of mature MPO are depicted in *gray*, *blue*, and *red*, respectively, together with the respective molar masses and number of amino acids. *B*, sequence of the primary translation product of human myeloperoxidase. Mature MPO is a homodimer with each monomer composed of a light (*blue*) and heavy chain (*red*). The signal peptide (*light gray*), the propeptide (*gray*), and a small peptide (depicted in *bold black letters*) are excised co- and posttranslationally. *Boxed* in *blue* and *red* are the alternative N termini of the light and heavy chains, respectively. Cysteine residues are depicted in *bold black* or *white*, and cysteine residue 319, which is responsible for dimer formation in mature MPO, is marked by the **#** symbol. Disulfides of MPO are shown by *black lines*. The cystine bridge in proMPO between Cys-158 and Cys-319 is shown as a *bold black line*. The *N*-glycosylation sites of both MPO forms are marked by an asterisk (*). ProMPO is a single peptide chain (Ala-49–Ser-745). The first resolved amino acid residues of the crystal structures of proMPO and MPO are depicted with *gray arrows*. Important catalytic residues are depicted in *bold*, and amino acid residues involved in the covalent heme to protein links are *underlined* and *bold*.

MPO biosynthesis encompasses a series of critical proteolytic processing steps during synthesis and intracellular trafficking. Fig. 1 depicts schematically the overall structure of monomeric proMPO (90 kDa) and homodimeric MPO (2× 74 kDa) and also provides the corresponding amino acid sequence of the primary translation product (15). In MPO biosynthesis the primary translational product (80 kDa, 745 aa) is converted into 90-kDa apoproMPO (700 aa) after cotranslational cleavage of the signal peptide and *en bloc N*-linked glycosylation followed by limited deglucosylation (16). ApoproMPO acquires heme in the endoplasmic reticulum (ER) thereby becoming enzymatically active 90-kDa promyeloperoxidase or proMPO (Fig. 1) (2, 17). Its export from the ER and subsequent processing require heme acquisition (17–19). ProMPO contains a 116-amino acid N-terminal pro-region that is required for its stability, as its deletion results in retention of proMPO in the ER and failure to undergo proteolytic processing to generate mature MPO (20), but its precise function and fate during MPO biosynthesis are unknown. A proconvertase eliminates the pro-region in a post-ER compartment producing a 74-kDa intermediate species that is subsequently further processed by cysteine proteases to the L-chain and H-chain (21) followed by dimerization. Most of the proMPO synthesized in the ER follows this proteolytic processing and targeting to azurophilic granules, but a substantial fraction is also constitutively secreted by normal bone marrow granulocyte precursors (2) and human myeloid cell lines (16, 22) as well as by heterologous MPO-expressing K562 cells (23), human embryonic kidney 293 (HEK) cells (24), or Chinese hamster ovary (CHO) cells (25).

Although several steps in MPO biosynthesis and processing have been elucidated, many unanswered questions remain, especially with respect to (i) the structure-function relationships of monomeric unprocessed proMPO *versus* mature dimeric MPO purified from azurophilic granules and (ii) the functional role of the propeptide. Here we explored the structure of proMPO, the importance of Cys-319 and Cys-319–Cys-319 bridge formation for stability, and the consequences of disrupting the dimerization of MPO. We present the high-resolution crystal structure of proMPO recombinantly produced by CHO cells and demonstrate how the flexible propeptide blocks dimerization by the formation of a disulfide bridge between Cys-158 from the propeptide and Cys-319. We show that the architecture of the heme cavity and the substrate access channel is already fully established at the proMPO stage and that the presence of the Cys-158–Cys-319 disulfide bond is essential for allowing proMPO to exit the ER and enter the Golgi apparatus for subsequent proteolytic processing. In the absence of the Cys-158–Cys-319 bridge, proMPO remains arrested in the ER, associated with the chaperones calnexin and calreticulin, and fails to undergo proteolytic processing to mature MPO.

## Results

### Biochemical properties and structural heterogeneities of proMPO and MPO

**Figure 2.**
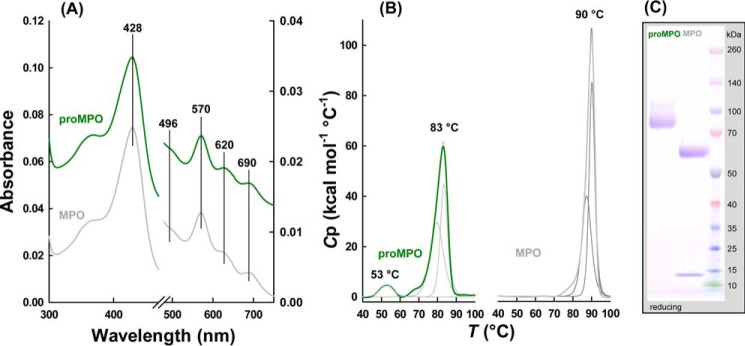
**Biochemical properties of monomeric proMPO and dimeric MPO.** *A*, UV-visible spectra of 8 μm (per heme) of proMPO (*green*) and MPO (*gray*) recorded in PBS. Spectra were shifted along the *y* axis for better visualization. *B*, thermal stability of proMPO and MPO evaluated by DSC. Thermal transitions of proMPO (*green*) and MPO (*gray*) were fitted to non-two-state equilibrium-unfolding models by the Levenberg-Marquardt nonlinear least squares method, and fits are depicted in *dark green* for proMPO and *dark gray* for MPO. *C*, SDS-PAGE of 2 μg of proMPO and MPO was resolved under reducing conditions on a 4–12% gradient gel.

Fig. 2*A* compares the electronic absorption spectra of the ferric forms of monomeric proMPO (*green line*) recombinantly produced in CHO cells with dimeric MPO (*gray line*) purified from neutrophils. The almost identical spectral features (Soret maximum at 428 nm and additional bands at 570, 620, and 690 nm) of proMPO and MPO clearly suggest the presence of a six-coordinated high-spin heme embedded in a very similar heme cavity (12). It is important to note that proMPO heterologously expressed in HEK293 cells exhibits almost identical biochemical properties (8).

The structural component of the present study utilized proMPO produced in CHO cells. The purity number (*A*_428_/*A*_280_) of the recombinant protein varied between 0.54 and 0.58, suggesting heme occupancy of >85%. Despite identical spectral features, proMPO and MPO exhibited significant differences in thermal stability and overall composition (see also Fig. 1). Monomeric 90-kDa proMPO showed one prominent endotherm upon unfolding with a *T_m_* value at 83 °C (Fig. 2*B*). The endotherm with *T_m_* at 53 °C can be assigned to unfolding of the fraction of heme-free protein, in agreement with the purity numbers (∼15% apoprotein), suggesting that the heme stabilizes proMPO. By comparison, homodimeric (mature) MPO comprises two 74-kDa protomers, each being composed of an L-chain (14.5 kDa) and an H-chain (59 kDa) (Fig. 2*C*). The thermal stability of the mature MPO (*T_m_* = 90 °C) was higher compared with that of proMPO (Fig. 2*B*), indicating that proteolytic processing and subsequent dimerization further stabilize the enzyme. It has to be mentioned that with both proMPO and MPO, deconvolution of the main endotherm suggests the presence of non-two-state transitions (*i.e.* the presence of at least one unfolding intermediate) but with close *T_m_* values (see corresponding fits in Fig. 2*B*).

Before undertaking X-ray structure determination, we analyzed the two purified MPO forms by mass spectrometry in order to probe the heterogeneity of the N termini of the respective polypeptide chains. Additionally, the N terminus of proMPO was tested by both Edman degradation and mass spectrometry. Upon using Edman degradation the N-terminal sequence A49-A-P-A-V-l-G-E-V-d-T was found and confirmed by mass spectrometry. Upon peptide mass mapping and analysis the peptide with the sequence A49-A-P-A-V-l-G-E-V-d-T-S-l-V-l-S-S-M-E-E-A-K was identified.

Interestingly, mass spectrometry revealed also that proteolytic maturation of MPO did not result in defined N termini of both the L- and the H-chain. In MPO purified from leukocytes, three different N termini for the L-chain were found, namely Gly-164 (identified peptide: ^164^GVTCPEQDKYR^174^), Val-165 (^165^VTCPEQDKYR^174^), and Thr-166 (^166^TCPEQDKYR^174^), respectively, resulting from the proteolytic elimination of the propeptide during maturation (compare with Fig. 1*A*). Another maturation step in MPO biosynthesis is the elimination of the hexapeptide ^273^ASFVTG^278^ resulting in the formation of the L- and H-chain (Fig. 1*A*). However, upon tryptic digestion of MPO and peptide analysis, it could be demonstrated also that the N terminus of the H-chain showed some heterogeneity, because three peptides (^277^TGVNCETSCVQQPPCFPLK^295^, ^278^GVNCETSCVQQPPCFPLK^295^, and ^279^VNCETSCVQQPPCFPLK^295^) were identified by mass spectrometric analysis of leukocyte MPO (compare with Fig. 1*B*). The amino acids ^273^ASFV^276^ could not be detected by MS analysis. This demonstrates that leukocyte MPO protomers were cleaved into the L- and H-chains by the elimination of tetra- (^273^ASFV^276^), penta- (^273^ASFVT^277^), or hexapeptides (^273^ASFVTG^278^).

### Crystal structure of proMPO

**Figure 3.**
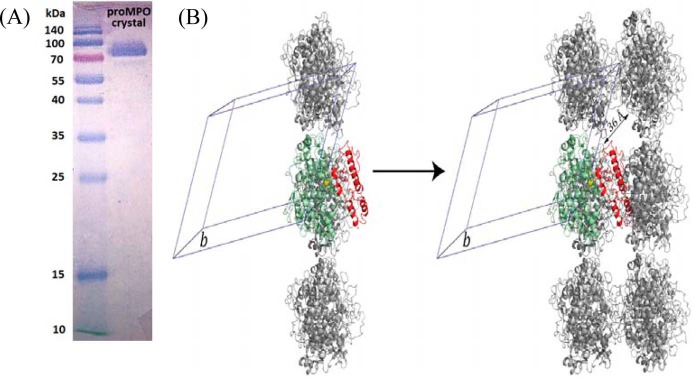
**Crystal packing.** *A*, SDS-PAGE of proMPO crystals. *B*, crystal packing. The core protein of proMPO of the asymmetric unit is presented in *green*, and the propeptide derived from modeling is shown in *red*. Symmetry-related molecules are presented in *gray*. The crystallographic axis *b* is indicated to help identify the solvent channels parallel to it. Additionally, one solvent-channel-width dimension is given.

Monoclinic proMPO crystals (space group C2) were grown with one proMPO molecule per asymmetric unit (Table 1). The structure was solved at 1.25-Å resolution by molecular replacement using the atomic coordinates of human leukocyte myeloperoxidase (PDB accession code 1MHL) as a search model. Interestingly, continuous electron density could only be seen for residues Gly-157 to Ala-744, with Gly-157–Val-165 belonging to the propeptide, whereas residues Ala-49–Ser-156 could not be modeled (compare with Fig. 1*B*). Analysis of the crystal packing boundaries showed a symmetry-related molecule in close contact with the propeptide region Gly-157–Val-165. If the propeptide were fully folded and in the position observed in the small-angle X-ray scattering (SAXS)-derived model (see below), the propeptide would clash with a symmetry-related molecule (Fig. 3*B*). However, solvent channels along crystallographic axis *b* are wide enough (38 × 36 Å) to accommodate the propeptide, which is about 29 Å wide in the folded state. Because an SDS-PAGE of the crystals demonstrated the presence of the full-length protein (Fig. 3*A*), this clearly suggests that the propeptide exhibits a high flexibility compared with the core of the protein (Thr-166–Ala-744). Analysis of the solvent content and Matthews coefficient (*V_M_*) for one molecule of proMPO per asymmetric unit yields 2.28 Å^3^/Da, well within the commonly observed range of 1.62 < *V_M_* < 3.53 Å^3^/Da).

**Table 1:**
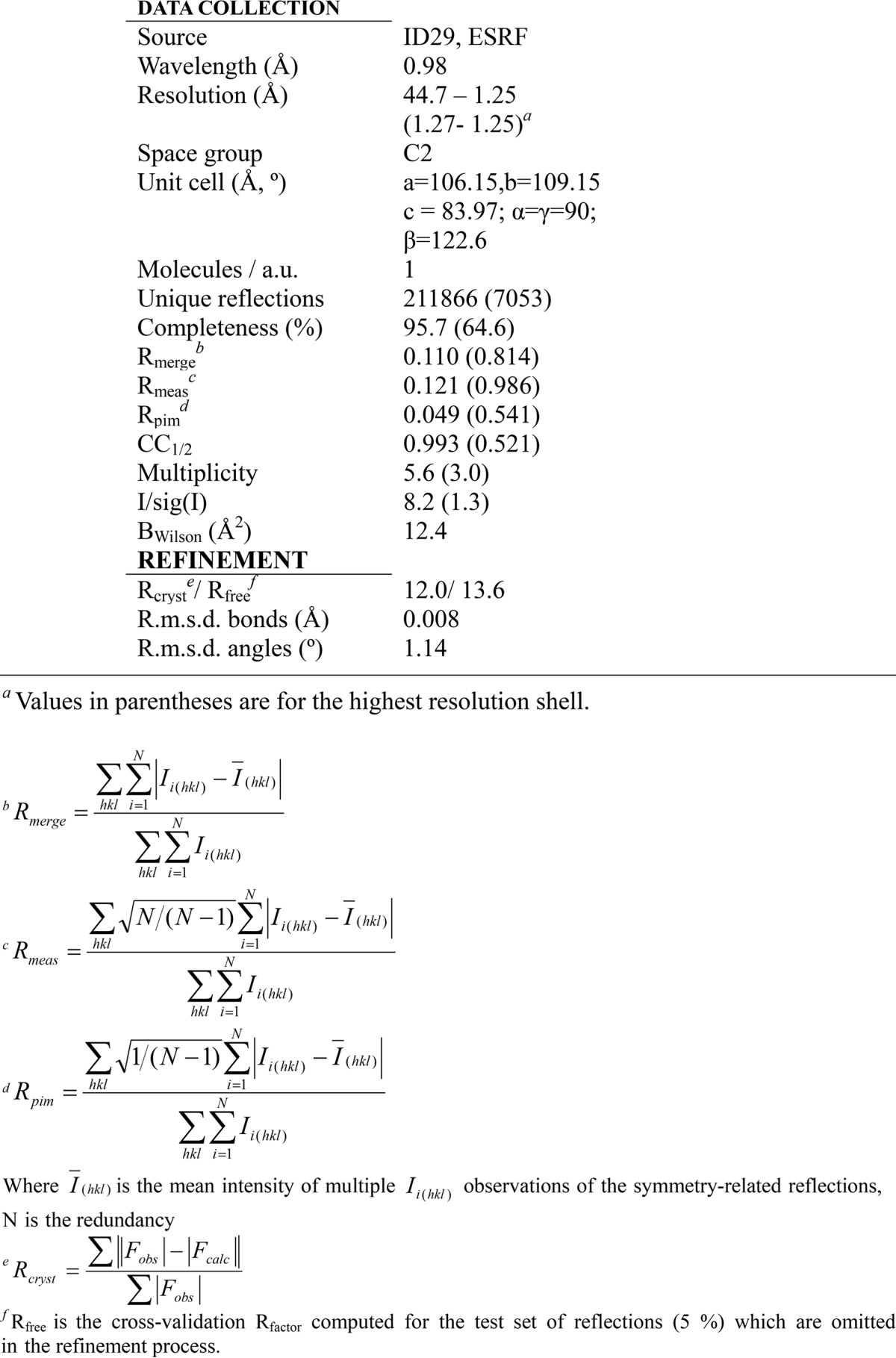
**Data collection and refinement statistics**

The greater flexibility of the propeptide compared with that of the core of proMPO is also reflected in the observed differences in thermal stability between proMPO and MPO (see above). Regarding the core structure of proMPO, the averaged root mean square deviation for the Cα atoms with respect to the 1MHL protomer structure of mature MPO revealed high similarity with a root mean square deviation of 0.49 Å over 576 superposed Cα atoms.

**Figure 4.**
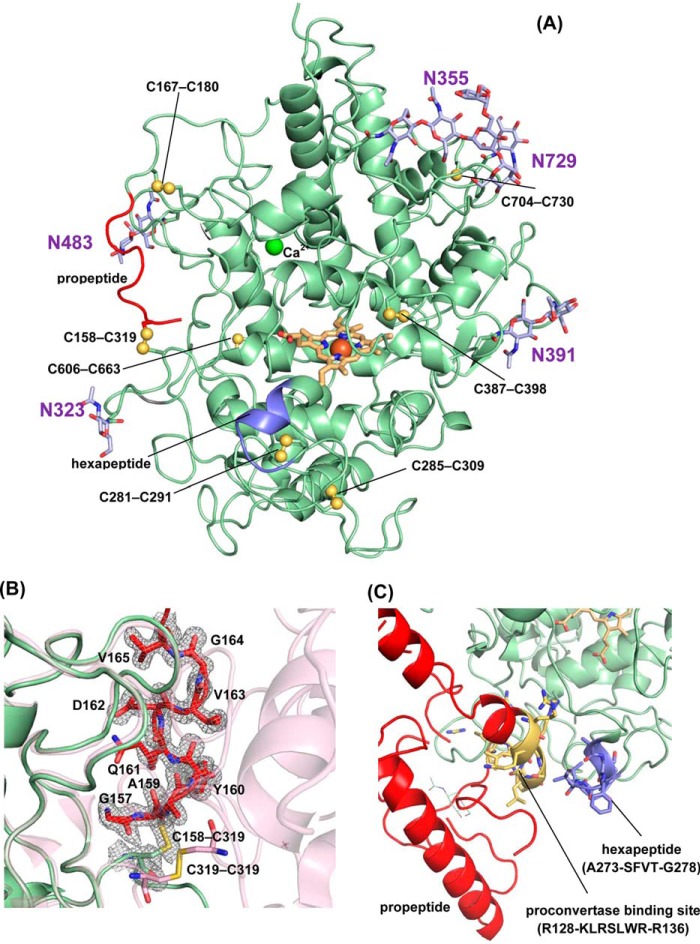
**Overall structure of proMPO.** *A*, crystal structure of recombinant proMPO. The disulfide bridges are depicted in *yellow*. In addition the *N*-glycosylation sites are shown in *light violet*. The segment of the propeptide visible in the electron density is shown in *red*, and the hexapeptide is shown in *blue. B*, close-up view of the C-terminal stretch of the propeptide (Gly-157–Val-165, shown in *red*) and the surrounding core-protein region, including the Cys-158–Cys-309 bridge, together with the 2 *F_o_* − *F_c_* electron density map contoured at the 1σ level. The second protomer in dimeric MPO, including the Cys-319–Cys-319 disulfide bridge, is depicted in *light pink* for comparison. *C*, close-up view of the area around the hexapeptide ^273^ASFVTG^278^ (*blue*) and neighboring proposed proconvertase-binding site (^128^RKLRS^132^) (highlighted in *yellow*). The rest of the propeptide is depicted in *red*.

The segment of the propeptide visible in the electron density of proMPO (Fig. 4*B*) is anchored to the core protein via a cystine bridge (Cys-158–Cys-319) together with polar and hydrophobic interactions, resulting in a buried surface area of 399 Å^2^. In particular, the guanidinium group of Arg-193 is engaged in two hydrogen bonds with the main chain carbonyl groups of Ala-159 and Gln-161, which stabilizes the main-chain conformation of the propeptide, whereas the side chain of Val-165 makes hydrophobic interactions with Met-179.

The structure of proMPO appeared to be highly glycosylated, showing five occupied *N*-glycosylation sites at Asn-323, Asn-355, Asn-391, Asn-483, and Asn-729, respectively (compare Fig. 4*A* with Fig. 1*A*), which confirms a recent comparative study on the *N*-glycan composition of MPO and proMPO (26). Clear electron density could be observed in all of them for the first *N*-linked GlcNac, with the exception of Asn-729 where electron density was poor due to either high flexibility or low occupancy of this site. The electron density indicates the presence of an additional sugar monomer in Asn-355, Asn-483, and Asn-391. Weak electron density could be observed for the branched GlcNac2-Man3 structure at Asn-355. Generally, the B-factors for the sugar atoms were relatively high, with an average value of 45.5 Å^2^ (average B-factor for the protein atoms is 17.5 Å^2^), which is consistent with the mobility and flexibility of glycan structures.

In mature MPO the glycan at Asn-323 (and to some extent sugar residues at Asn-483) contributes mainly to the interface between the two protomers (schematically shown in Fig. 1*A*) and thus stabilizes the homodimeric structure (5, 7, 8). The hybrid model (see below) suggested that Asn-323 was embedded in the interface between the propeptide and the core protein. The putative *N*-glycosylation site at the propeptide (Asn-139) could not be verified here because of the lack of electron density. However, recent mass spectrometric analysis showed that Asn-139 of the recombinant form is glycosylated (26). Moreover, the hybrid model and the SAXS data (see below) suggest that Asn-139 is in proximity to Asn-323, which might indicate that the respective glycan chains interact. By contrast, in proMPO the glycan at Asn-483 did not participate to the interface between the propeptide and the core protein.

Furthermore, the crystal structure of proMPO displayed the presence of six disulfide intrachain bridges, namely Cys-167–Cys-180, Cys-281–Cys-291, Cys-285–Cys-309, Cys-387–Cys-398, Cys-606–Cys-663, and Cys-704–Cys-730, respectively (Fig. 4*A*; compare with Fig. 1*B*). The same disulfide bridges are found in mature MPO, with Cys-167–Cys-180 located in the L-chain and the remaining disulfide bridges belong to the H-chain of leukocyte MPO. However, most interestingly, Cys-319 of proMPO, which is known to form an interchain disulfide bridge between the H-chains of the symmetry-related halves (74 kDa) in dimeric MPO (compare with Fig. 1*A*) (5, 7, 8), formed a disulfide bridge with Cys-158 of the propeptide. Fig. 4*B* depicts a close-up of the C-terminal stretch of the propeptide (Gly-157–Val-165) and the neighboring core-protein region including the Cys-158–Cys-309 intrachain bridge in comparison with the interface between the two protomers in mature MPO, which are linked by the Cys-319–Cys-319 interchain bridge.

As outlined above, one proteolytic maturation step of proMPO includes the excision of a tetra-, penta-, or hexapeptide resulting in the creation of the L- and H-chain. It has been proposed that the hydrolytic cleavage is mediated by a cysteine protease located in the Golgi network (21). The crystal structure of proMPO shows that the stretch ^273^ASFVTG^278^ was part of a surface-exposed α-helix loop segment (Fig. 4*C*) with all six amino acids surface exposed (accessible surface areas follow the hierarchy Phe-275 > Gly-278 > Val-276 > Ala-273 > Ser-274 > Thr-277) (Fig. 4*C*). Interestingly, the hybrid model (see below) indicates that Lys-129 and Ser-132 of the helix formed by the residues Leu-125–Ser-132 of the propeptide interact with this region of the core protein (Fig. 4*C*). Note that ^128^RKLRS^132^ is part of the proposed proconvertase-binding site (see below). This suggests that the propeptide not only hinders dimerization by blocking Cys-319 and embedding the glycan at Asn-323 in the propeptide-core protein interface but also hinders access of the cysteine protease to the surface-exposed ^273^ASFVTG^278^ region.

**Figure 5.**
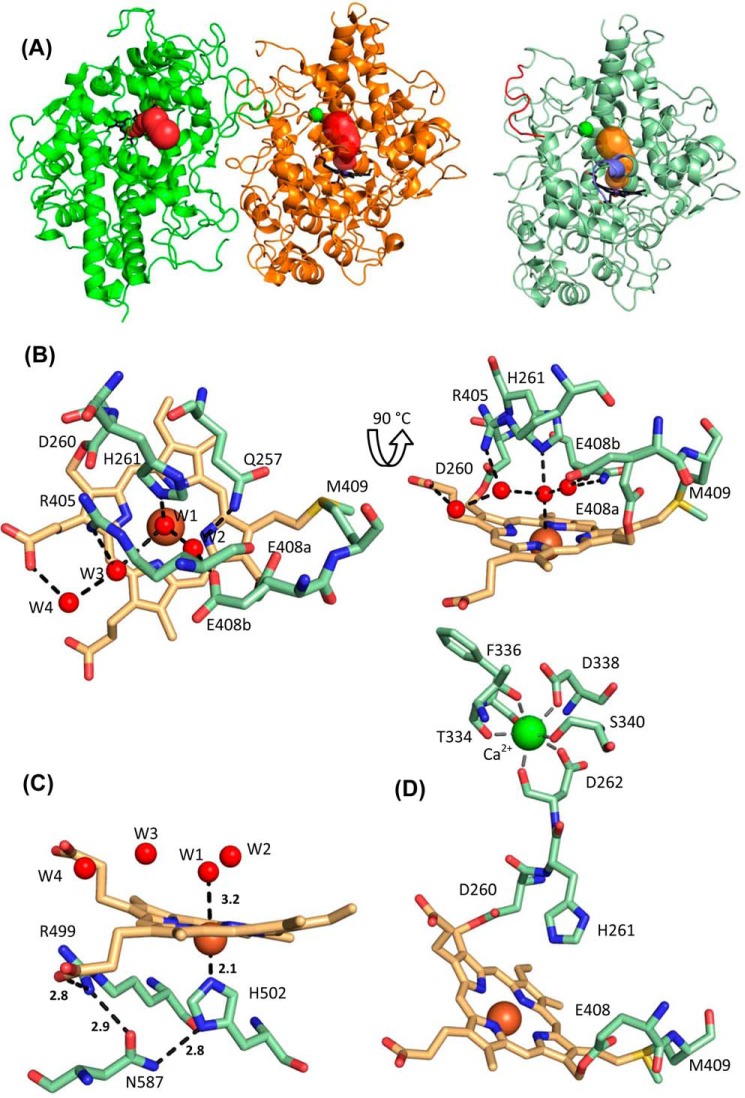
**Heme cavity architecture of proMPO.** *A*, substrate-access channels in mature MPO (*left panel*) and proMPO (*right panel*). The segment of the propeptide visible in the electron density map is shown in *red*. The channels were calculated using the tool CAVER 3.0 (57). For better orientation, the prosthetic group is highlighted in *bold black. B*, distal heme cavity and H-bonding network of proMPO. The catalytic residues His-261, Arg-405, and Gln-257 including W1–W4 and H-bondings are shown. The residues Asp-260, Glu-408, and Met-409 are involved in heme-to-protein linkages. The split conformation of Glu-408 is designated *E408a* and *E408b. C*, prosthetic group and proximal ligand His-502 together with the interaction network His-502–Asn-587–Arg-499 and the propionate of pyrrole ring D (distances are given in Å). *D*, calcium-binding site in proMPO.

Importantly, the propeptide of proMPO did not interfere with substrate accessibility to the heme cavity. Fig. 5*A* schematically presents and compares the main access channels in the two protomers of MPO and proMPO. The length of the channels in MPO and proMPO (20.3 and 20.7 Å), the bottleneck radius (0.97 and 1.03 Å), and the curvature (length over distance, 1.29 and 1.34), respectively, were almost identical. Moreover, the architecture of the heme cavity was almost superimposable. In both MPO and proMPO the heme group is posttranslationally modified and covalently linked with the protein. The ester bond between Asp-260 and a hydroxymethyl group on pyrrole ring C was present, as was the sulfonium ion linkage between the β-carbon of the vinyl group on pyrrole ring A and the sulfur atom of Met-409 (Fig. 5*B*). Similar to leukocyte MPO (8), the side chain of Glu-408 had a low electron density, indicating high mobility and suggesting that the ester bond was present only in a fraction of the population of protein molecules (Fig. 5*B*). Similar to MPO, the electron density at the methyl substituent of pyrrole ring A in proMPO suggested hydroxylation. As a consequence of these modifications, pyrrole ring A and, to a lesser extent, ring C were tilted toward the distal side, resulting in a bow-shaped heme structure.

The prominent catalytic distal residues in proMPO are His-261 and Arg-405, which are important in heterolytic cleavage of hydrogen peroxide during compound I formation, and Gln-257, which is involved in halide binding. The distal heme cavity contained four water molecules (W1–W4), which formed hydrogen bonds with His-261, Arg-405, and Gln-257, and to the heme pyrrole ring C propionate as well as between themselves. The distal His-261 was hydrogen-bonded to W1, which is positioned approximately midway between the histidine nitrogen and the iron (Fig. 5*B*). Overall, this H-bonding network was almost identical to that in mature MPO.

The proximal heme-iron ligand, His-502, interacted with the amine group of the side chain of Asn-587, whereas the carbonyl group of Asn-587 interacted with the guanidinium group of Arg-499. Furthermore, Arg-499 formed a salt bridge with the heme propionic group at pyrrole ring D (Fig. 5*B*). This interaction, identical to that in leukocyte MPO (8), requires an anionic His-502, which was facilitated by lowering the p*K_a_* in His-502 as a result of its coordination with the heme iron. The respective distances in proMPO and MPO were almost identical (Fig. 5*C*). The structural similarities between proMPO and dimeric MPO apply also to the distal Ca^2+^-binding site with its typical pentagonal bipyramidal coordination provided by residues Asp-262, Thr-334, Phe-336, Asp-338, and Ser-340 (Fig. 5*D*).

### Hybrid model of proMPO

Because the structure of a significant portion (Ala-49–Ser-156) of the N-terminal propeptide in proMPO could not be resolved in electron density maps, SAXS analysis was performed in addition (see below). For the propeptide domain an α-helical structural model was calculated using the PHYRE2 Protein Fold Recognition Server (Structural Bioinformatics Group, Imperial College, London). The confidence level of the predicted structure was at most 76%, covering the main helical regions (Leu-54–Ile-84 and Ser-90–Val-121) and suggesting that alternative conformations are not unlikely.

The residues Gly-157–Val-163 of this model structure were superposed with the corresponding resolved N terminus in the X-ray structure, thus maintaining the Cys-158–Cys-319 disulfide bridge. Finally, this hybrid model was energy-minimized with MOE (molecular operating environment) (27) and the Amber99 (28) force field and used to fit the experimental SAXS data of proMPO. Eight of the 697 residues in the resulting model are outside the allowed regions in the Ramachandran plot (7 of the 107 modeled propeptide residues), further suggesting that alternative conformations of the propeptide are not unlikely.

**Figure 6.**
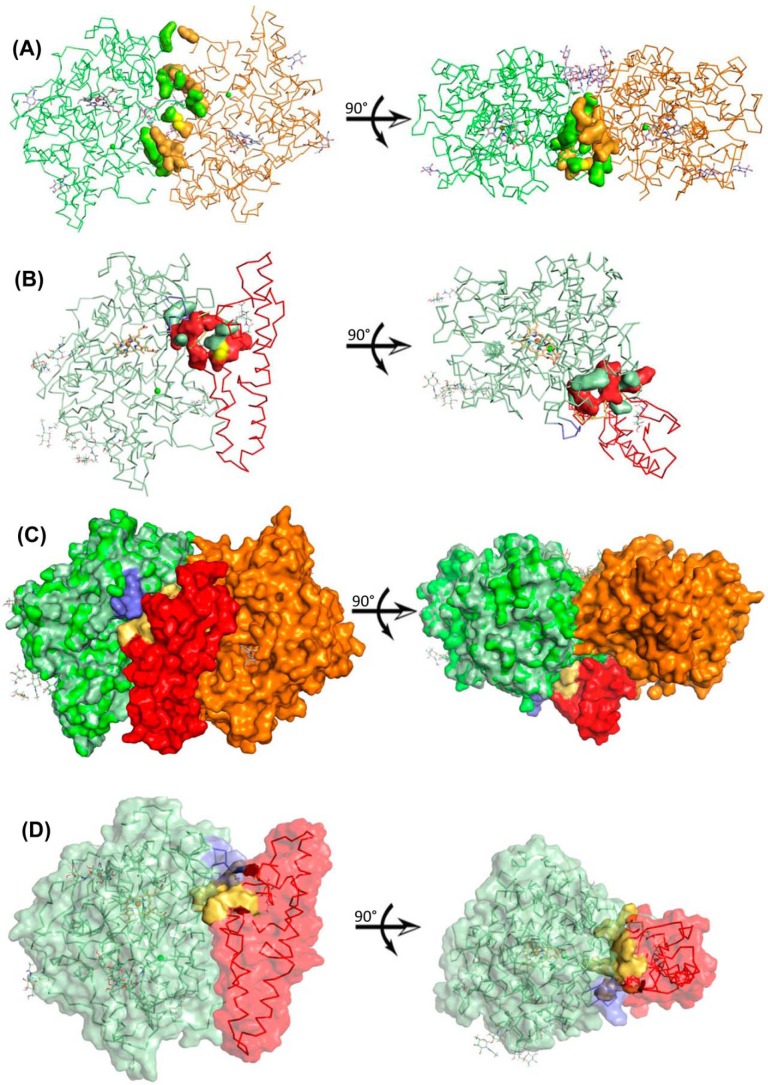
**Hybrid-model structure of proMPO.** *A*, interface between the two identical protomers (chain A depicted as a *green ribbon* and chain B as an *orange ribbon*) of mature MPO (PDB accession code 3F9P). Residues involved in noncovalent interactions are presented as space-filling models (Arg-184–Glu-202, Ser-185–Ala-201, Thr-187–Gly-204, Arg-193–Asn-323, Arg-193–Ile-324, Ala-201–Ser-185, Glu-202–Arg-184, Gly-204–Thr-187, Lys-218–Glu-169, Cys-319–Cys-319, Asn-323–Arg-193, and Ile-324–Arg-193). *B*, interface between the propeptide (*red*) and the core protein of proMPO (*pale green*). Residues involved in noncovalent interactions are presented as space-filling amino acids (Glu-127–Cys-316, Arg-131–Arg-314, Arg-135–Gly-321, Lys-154–Thr-325, Ser-155–Cys-319, and Cys-158–Cys-319). Sulfur atoms in Cys-158–Cys-319 are shown in *yellow*. Residues belonging to the propeptide are shown in *red. C*, surface representation of the overlay of mature MPO and propeptide of proMPO in the colors described above. The hexapeptide is depicted in *blue. A–C* are represented from identical angles. *D*, proposed binding site (^128^RRKLRSLWR^136^) of proconvertase in proMPO (*yellow* surface). The propeptide is depicted in *red* and the core protein of proMPO in *pale green*.

Fig. 6 compares the interface between the two protomers in mature MPO (basis is the X-ray structure with PDB accession code 1MHL) with that between the propeptide and the core protein in proMPO (basis: hybrid model). The residues involved in noncovalent interactions in the interface between the two protomers of mature MPO (Arg-184–Glu-202, Ser-18–Ala-201, Thr-187–Gly-204, Arg-193–Asn-323, Arg-193–Ile-324, Ala-201–Ser-185, Glu-202–Arg-184, Gly-204–Thr-187, Lys-218–Glu-169, Cys-319–Cys-319, Asn-323–Arg-193, and Ile-324–Arg-193) (Fig. 5*A*) are completely different from the residues involved in noncovalent interactions between the propeptide and the core of the protein in proMPO (Glu-127–Cys-316, Arg-131–Arg-314, Arg-135–Gly-321, Lys-15–Thr-325, Ser-155–Cys-319, Cys-158–Cys-319, Ala-159–Arg-191, Gln-161–Arg-191, and Val-165-Met-179, with the latter three interactions also seen in the crystal structure) (Fig. 6*B*). A comparison of Fig. 6, *A* and *B*, clearly demonstrates that the interface in mature MPO exhibits significantly more noncovalent interactions that contribute to its higher thermal stability compared with proMPO. This effect results in a 1332-Å^2^ buried surface area upon MPO dimer formation compared with 399 Å^2^ between the propeptide and the core protein in proMPO. Moreover, the two interfaces are not topologically equivalent, as illustrated in Fig. 6*C*, which shows a surface representation of the overlay of mature MPO and the propeptide of proMPO.

The most important proteolytic maturation step in MPO biosynthesis concerns the cleavage of the propeptide. It has been demonstrated that an inhibitor of subtilisin-like proteinases blocks cleavage of the propeptide in a post-ER compartment; by mutational studies the positively charged proconvertase target sequence ^128^RKLRSLWRR^136^ was identified (21). The hybrid-model structure suggests that Arg-128, Lys-129, Leu-133, Trp-134, and Arg-136 have a highly accessible surface area for interaction with the proconvertase (Fig. 6*D*).

### Solution structures of proMPO and MPO

**Figure 7.**
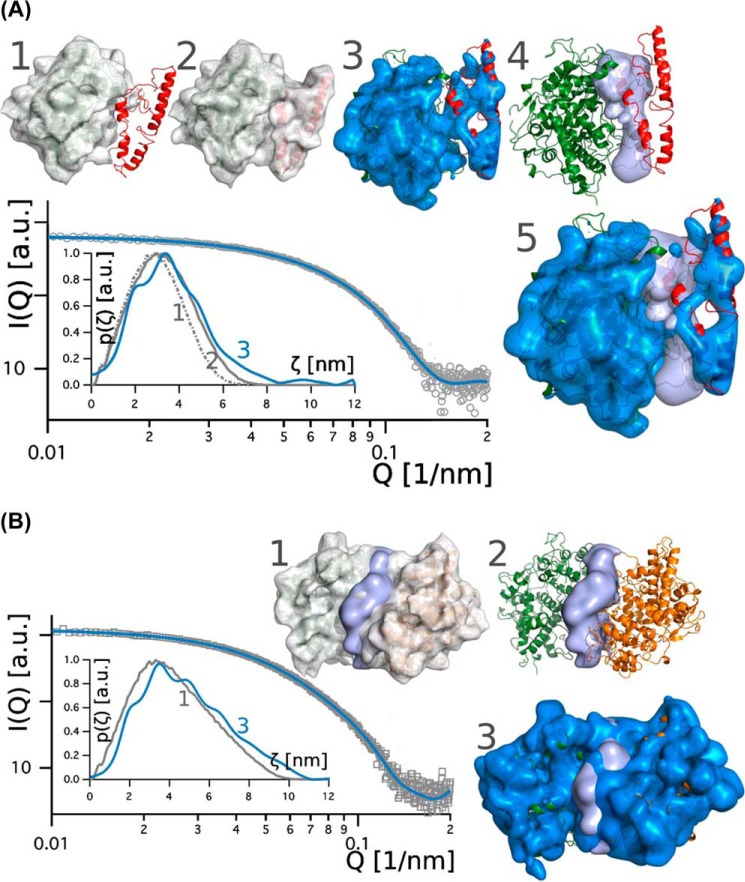
**Solution structures of proMPO and dimeric leukocyte MPO obtained by SAXS.** *A*, promyeloperoxidase: plot of scattering intensity I(Q) (*gray circles*) and fit (*blue line*) *versus* scattering vector. The *inset* shows pair-density distributions *p*(ζ) computed for: the proMPO crystal structure (*dotted gray line 1*); the hybrid model, which contains the propeptide (*gray line 2*); and *p*(ζ) computed from the scattering intensity (*solid blue line 3*). The corresponding surface models (depicted in *gray*) are superimposed onto the schematic representation of the crystal structure (*model 1*) and the hybrid model structure (*model 2*) of proMPO. The *blue* surfaces in *models 3* and *5* comprise scattering sites that contribute to I(Q); χ2 of the model is 0.91. The *light blue* surface comprises residues that potentially contribute to the interface between the propeptide and the compact rest of the protein. In *model 4* the secondary structural elements of the core protein (*green*) and the propeptide (*red*) are shown. *B*, mature dimeric leukocyte MPO: plot of scattering intensity I(Q) (*gray circles*) *versus* scattering vector including fit (*blue line*). In the *inset* we compared the pair-density distribution *p*(ζ) computed for the background-corrected data (*blue line*) and the MPO crystal structure (*gray line*). The corresponding surface models are depicted in *gray* (*model 1*) and *blue* (*model 3*); χ2 of the model is 0.90. The *light blue* surface comprises residues that potentially contribute to the interface of the dimer. In *model 2* the secondary structural elements of the two protomers of MPO are depicted in *green* and *orange. a. u.,* arbitrary units.

Next we performed a SAXS analysis of proMPO and MPO in solution (50 mm PBS, pH 7.4). Fig. 7*A* shows the scattering intensity (I) for proMPO plotted *versus* the scattering vector (Q), with *gray circles* representing experimental data and the *blue line* the corresponding fit. The *inset* (Fig. 7*A*) shows the pair-density distribution *p*(ζ) computed for the crystal structure of proMPO (*dotted gray line 1*), for the hybrid model (*gray line 2*), and for the background-corrected scattering data (*blue line 3*). The pair-density distribution computed for the hybrid structure showed only small deviations from *p*(ζ) as deduced from the background-corrected scattering data, thus increasing confidence in the propeptide model (the agreement between the computed and experimental *p*(ζ) is as good for the dimeric MPO structure; see below). Differences were seen at distances >4 nm with a more pronounced tailing of *p*(ζ) computed for the experimental data.

In addition Fig. 7*A* depicts the corresponding calculated surface models. The pair-density distribution computed for the hybrid-model structure (Fig. 7*A*, *gray model 2*) was refined, leading to volume *models 3* and *5* with *blue* surfaces representing the (rigid) regions of the protein that contribute to the scattering signal with weights >1; The *light blue* surface comprises residues that potentially contribute to the interface between the propeptide and the compact rest of the protein. *Model 4* (Fig. 7*A*) depicts the secondary structural elements of the hybrid structure of proMPO for orientation. Upon inspection of these data it is evident that the scattering contrast in the propeptide and the interface region was low, suggesting less compactness of the propeptide and a loose interaction with the rest of the protein, which may also explain the absence of defined electron density in the majority of the propeptide in the X-ray structure of proMPO.

For comparison, leukocyte MPO was analyzed under identical conditions (50 mm PBS, pH 7.4). The *inset* in Fig. 7*B* shows the pair-density distribution *p*(ζ) computed for the background-corrected scattering data (*blue line 3*) and the crystal structure of MPO (*gray line 1*) (PDB accession code 3F9P). Both *p*(ζ) values are scale-invariant although differing slightly in corrugation. The corresponding surface models (Fig. 7*B*) are shown in *gray* (*model 1*) and *blue* (*model 3*), with the *light blue* surface comprising residues that potentially contribute to the interface of the dimer. *Model 2* (Fig. 7*B*) depicts the secondary structural elements of the crystal structure of the two protomers of MPO for orientation. It is evident that the interaction between the two protomers in MPO was significantly tighter than between the propeptide and the rest of the protein in proMPO.

**Figure 8.**
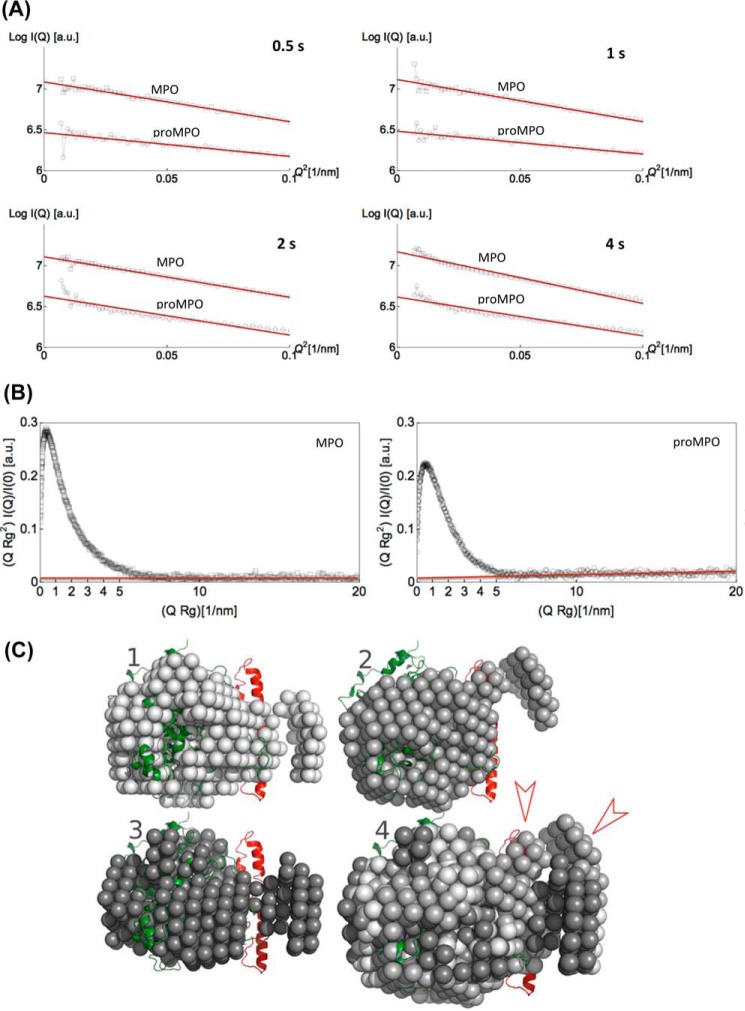
**Guinier and normalized Kratky plots of dimeric MPO and proMPO and bead models of proMPO.** *A*, Guinier plots. The logarithm of scattering intensity (lnI(Q)) of dimeric mature MPO (*gray squares*) and proMPO (*gray circles*) are given as a function of Q2. Four different times of data acquisition (0.5, 1, 2, and 4 s) are compared. For leukocyte MPO, nonlinearity is seen after 4 s, whereas for proMPO, nonlinear effects are seen already after 2 s of beam exposure. Nonlinearity is taken as an indication of the onset of radiation damage. *B*, normalized Kratky plot given as a function of the radii of gyration *R_g_* and extrapolated scattering intensity I(Q) for dimeric mature MPO (*gray squares*) and proMPO (*gray circles*). Note the difference in slope of the baseline (*red line*). Although the baseline for leukocyte MPO is constant, the baseline for proMPO shows a positive slope, which is an indication of the soft binding of the propeptide to the core of proMPO. *a. u.,* arbitrary units. *C*, *bead* models of proMPO derived from data analysis using the software ATSAS 2.7.2. We computed three different models using the program package ATSAS 2.7.2. For computation of the bead models, we used the pair-density distributions depicted in Fig. 6*A*. These bead models were aligned by PyMOL and superimposed in *model 4*.

Fig. 8 compares the Guinier plots for four different times of data acquisition (0.5, 1, 3, and 4 s), demonstrating that up to 2 s of beam-exposure scattering data is free of radiation damage. From the Guinier plots, the radii of gyration for dimeric MPO (3.8 nm) and monomeric proMPO (2.9 nm) were obtained, again demonstrating that the latter is less compact. Additionally, we computed normalized Kratky plots (Fig. 8*B*). Both proteins exhibited shapes characteristic of folded proteins. However, although the baseline for MPO is constant, the slope is positive for proMPO, which indicates increased flexibility in the latter.

Moreover, we computed a set of bead models for proMPO accessed by the program ATSAS (Fig. 8*C*) (29). We changed the maximum particle diameter between 10 and 10.5 nm and constructed therefrom three different bead models (Fig. 8*C*, *models 1–3*). In *model 4* we aligned the bead models using PyMOL, and finally, we manually superimposed the hybrid model in a schematic representation (Fig. 8*C*). The propeptide appears partially structured but flexible, and the two clusters of beads (Fig. 8*C*, *red arrowheads*) indicate a compact and less compact configuration. This is in line with findings of the normalized Kratky plot. In summary, the SAXS analysis clearly suggests that the propeptide is structured (as suggested by the hybrid model) but flexible, thus leading to incoherent scattering and missing electron density in the crystal structure.

### Biosynthesis and enzymatic activity of the mutant proteins C319A, C158A, and C158A/C319A

**Figure 9.**
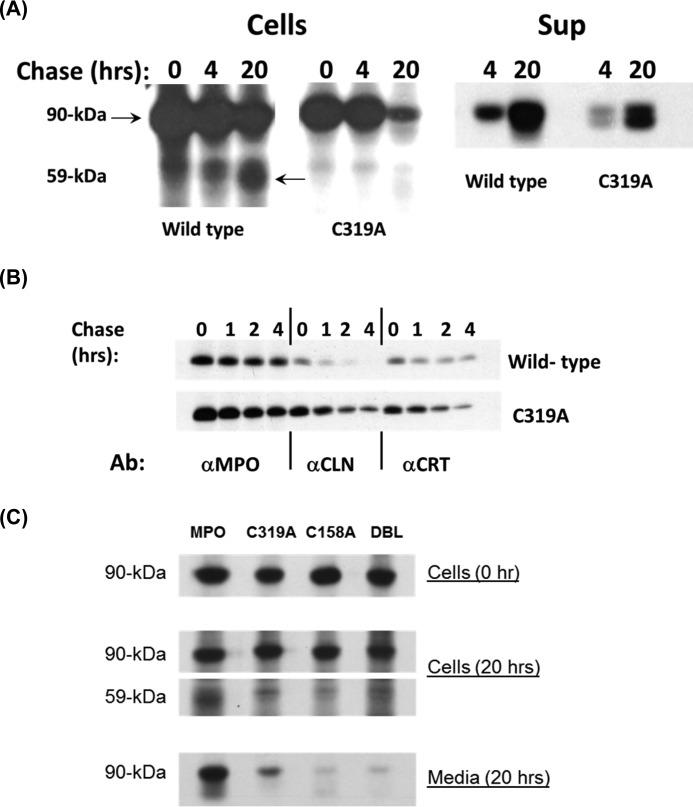
**Biosynthesis of mutants C319A and C158A and double mutant C158A/C319A.** *A*, transfectants expressing wild-type MPO or C319A were radiolabeled biosynthetically and chased for 0–20 h. Cell lysates (*Cells*) and supernatants (*Sup*) were immunoprecipitated for MPO-related proteins, including the 90-kDa precursors (apo- and proMPO) and the 59-kDa heavy subunit of mature MPO. *B*, stable transfectants expressing wild-type MPO or C319A were radiolabeled biosynthetically and chased for 0–4 h. Lysates were immunoprecipitated for MPO (α*MPO*) or sequentially immunoprecipitated with αCLN → αMPO to recover CLN-associated MPO (α*CLN*) or with αCRT → αMPO to recover CRT-associated MPO (α*CRT*). A representative of three to five independent experiments is shown. *C*, transfectants expressing wild-type MPO, C319A, C158A, or C319A/C158A (*DBL*) were radiolabeled biosynthetically and chased for 0–20 h. Cell lysates (*Cells*) and supernatants (*Media*) were immunoprecipitated for MPO-related proteins, including the 90-kDa precursors (apo- and proMPO) and the 59-kDa heavy subunit of mature MPO. A representative of four to six independent experiments is shown.

In mature MPO the interchain disulfide bond bridges the two heavy-chain subunits (5, 7, 8). Our structural data demonstrated that in proMPO, Cys-319 existed in an intrachain disulfide linkage with Cys-158 of the propeptide region. To analyze the importance of dimerization to biosynthesis and the activity of mature MPO, we generated stable HEK transfectants expressing the mutant C319A. The mutant protein did not undergo significant proteolytic processing to form mature dimeric MPO even after prolonged chase (Fig. 9*A*). However, a fraction of the mutant precursor entered the secretory pathway and appeared in culture media, as occurred with transfectants expressing wild-type MPO (Fig. 9*A*). Fractionation of biosynthetically radiolabeled transfectants on sucrose-density gradients demonstrated a difference in the distribution of precursors in cells expressing wild-type or mutant protein. After a 4-h chase, wild-type MPO species were distributed in two peaks (fractions 8–9 and fraction 16), whereas MPO-related immunoreactivity for C319A was found only in fractions 8–9 (not shown), fractions that we had shown previously to be enriched for ER (30). These data suggested that the mutant C319A was retained in the ER and did not gain access to the cellular compartments where proteolytic processing of proMPO normally occurs (17, 22).

Early in its biosynthesis, MPO precursors apoMPO and proMPO associate transiently with molecular chaperones calreticulin (CRT) and calnexin (CLN) (23, 31). To determine how the mutant C319A interacts with ER chaperones, we immunoprecipitated CRT- and CLN-associated MPO precursors from transfectants expressing wild-type or mutant MPO. The association of CLN with C319A was especially prolonged in comparison with that seen for wild-type MPO (Fig. 9*B*). At 4 h of chase, 2.5% of the wild-type proMPO remained associated with CLN, whereas 34.5% of the mutant protein was in a complex.

Additionally, we created HEK cell lines expressing targeted mutations at Cys-158 as well as both Cys-158 and Cys-319. The biosynthesis of the respective mutant proteins was compromised in a similar fashion in both cell lines, with dramatic effects both on proteolytic processing to mature MPO subunits and on the secretion of proMPO into the culture medium (Fig. 9*C*). For example, the ratio of the 90-kDa MPO precursor to the 59-kDa heavy-chain subunit of mature MPO, a calculation that correlates with efficient processing of proMPO (21), was increased in C319A, C158A, and C158/C319A relative to that in cells expressing wild-type MPO (Table 2). In addition, cells expressing the mutant forms of MPO secreted significantly less proMPO than did transfectants expressing normal MPO. Like the C319A proMPO, the precursors of C158A and C319A/C158A exhibited prolonged associations with CLN (data not shown). Taken together, the failure to form a disulfide bond between Cys-158 and Cys-319 in the MPO precursor in the ER disrupted both normal proteolytic processing and the secretion of mutant proMPO.

**Table 2:** **Processing of wild-type and mutant proMPO** Stably transfected HEK cells expressing wild-type or mutant MPO were radiolabeled biosynthetically, and MPO-related proteins were immunoprecipitated immediately after labeling (at 0 h) and 20 h after chase. Immunoprecipitates were separated by SDS-PAGE. The amount of immunoprecipitated 90- and 59-kDa MPO-related protein at 20 h of chase and the amount of MPO-related protein secreted was quantitated using a phosphorimaging device.

Source	90 kDa:59 kDa (*n*)*^a^*	% Secreted*^b^*
Wild-type MPO	0.72 ± 0.08 (13)	100
C319A	1.96 ± 0.27 (5)	28.8 ± 7.9
C158A	1.62 ± 0.24 (6)	5.1 ± 2.0
C158A/C319A	1.64 ± 0.30 (4)	11.9 ± 1.3

*^a^* The ratio of precursor to mature MPO (90 kDa:59 kDa) is shown as mean ± S.E. for *n* number of experiments. The p values for the differences of 90 kDa:59 kDa for C319A, C158A, and C158A/C319A *versus* that of wild-type MPO are .0001, .0003, and .0009, respectively.

*^b^* The percent MPO-related protein normalized to that secreted by HEK-MPO cells is shown as the mean ± S.E. for four separate experiments.

During the processing of normal MPO, apoproMPO acquires heme in the ER, and the resultant proMPO exits the ER, undergoes proteolytic processing, and after a prolonged period (32, 33) forms homodimeric MPO (reviewed in Ref. 15). Previous studies have linked heme acquisition by MPO precursors to the egress of proMPO from ER and subsequent maturation. Pharmacologic inhibition of heme synthesis by succinyl acetone results in a maturation arrest in MPO biosynthesis, with accumulation of enzymatically inactive apoproMPO (17, 18). Consequently, we reasoned that the formation of the Cys-158–Cys-319 disulfide bond might influence efficient heme incorporation into apoproMPO and the generation of proMPO. To assess heme acquisition by mutant precursors, we measured the enzymatic activity of lysates of HEK cells and HEK transfectants expressing wild-type MPO and the three mutants. Given that both peroxidase activity and chlorinating activity each depends on the functional integrity of the heme center and that the inability to support peroxidation would preclude the capacity to chlorinate, we elected to compare the peroxidase activity of the mutants with that of wild-type MPO. Compared with the activity of HEK transfectants expressing wild-type MPO, the activities of the mutant cell lines were significantly reduced. Whereas the activities of C158A and C158A/C319A were not significantly higher than that of wild-type HEK cells, which lack an endogenous peroxidase, lysates of C319A possessed peroxidase activity, albeit significantly less than that of transfectants expressing normal MPO (Table 3).

**Table 3:** **Peroxidase activity of wild-type and mutant cell-associated MPO and secreted proMPO species** Cell lysates and conditioned culture media from stably transfected HEK cells expressing wild-type or mutant MPO were assayed for peroxidase activity. Lysates of 5 × 10^5^ cultured cells and 10 μg of column-enriched culture media were used in the assays, except for spent media from transfectants expressing normal MPO (*), where 1 μg was used. Under the assay conditions, the Δ*A*_650_ value for 2 pmol of purified MPO was 0.190 ± 0.007 (*n* = 9).

Source	Cell-associated MPO (*n*)*^a^*	Secreted proMPO (*n*)*^a^*
HEK cells	0.043 ± 0.001 (9)	0.046 ± 0.001 (3)
Wild-type MPO	0.346 ± 0.009 (9)	1.264 ± 0.173* (3)
C319A	0.097 ± 0.004 (6)	0.648 ± 0.038 (3)
C158A	0.043 ± 0.001 (6)	0.058 ± 0.005 (3)
C158/C319A	0.048 ± 0.001 (3)	0.517 ± 0.089 (3)

*^a^* The results are given as mean ± S.E. *n* = 3–9 for studies of cell-associated activity and n = 3 for studies of secreted proMPO. The peroxidase activity of lysates from C158A and C158/C319A was not significantly different from that of untransfected HEK cells. The activity of C319A differed from that of the untransfected cells (*p* = .007).

During MPO biosynthesis in myeloid precursors from bone marrow, myeloid cell lines, or transfectants expressing heterologous MPO, a fraction of the newly made proMPO is constitutively released into the culture medium (2, 16, 20, 32, 34–37). Secreted proMPO exhibits the same specific activity as intracellular proMPO or mature MPO (38), thus providing an additional insight into the functional status of the respective pro-forms of the mutants. Secreted proMPO was isolated from spent media using cation chromatography as done previously (21), and peroxidase activity was measured (Table 3). Overall, the activity of supernatants from all mutants was less than that of normal proMPO. Because this method recovers all cationic proteins in spent media and provides material enriched for proMPO, we performed immunoblots of samples eluted from the beads to assess the fraction of MPO-related proteins in supernatant. Compared with the amount of proMPO secreted by transfectants expressing normal MPO, all mutants were reduced; C319A, C158A, and C158A/C319A secreted 8.4 ± 0.8, 7.5 ± 0.2, and 5.9 ± 0.6% (*n* = 4 for each), respectively, of the amount of normal proMPO. Even when corrected for the relative amount of MPO-related protein, the peroxidase activity of secreted proMPO from all three mutants was depressed compared with normal proMPO, with C158A being the most profoundly decreased (Table 3).

## Discussion

The structural and functional studies presented here elucidate several previously unknown features of MPO biosynthesis. Myeloperoxidase gene transcription is limited to early myeloid precursors in the bone marrow, when MPO is synthesized and stored in azurophilic granules for subsequent release from stimulated neutrophils. With the help of the atypically long 45-aa N-terminal signal peptide, the primary translation product channels into the ER, where it undergoes cotranslational cleavage of the signal peptide and *en bloc N*-linked glycosylation with two *N*-acetylglucosamine residues at the base of the six-glycan chains to yield apoproMPO. ApoproMPO has a very long half-life in the ER. The added oligosaccharides contribute to interactions with the ER molecular chaperones CRT, CLN, and ERp57 (23, 31), which generally promote proper folding and quality control in glycoprotein biosynthesis (39). In addition to the presence of *N*-linked oligosaccharides, the overall conformation of the apoproMPO influences its interaction with the ER chaperones, as demonstrated by the impact of disruption of the Cys-158–Cys-319 disulfide bridge on association with CLN (Fig. 9*B*). The compactness and noncovalent interactions at the interface between the propeptide and the core protein in proMPO is less pronounced compared with the interface in mature homodimeric MPO, and elimination of the Cys-158–Cys-319 disulfide bridge in proMPO will even boost this difference and promote (partial) unfolding. The same holds true for apoproMPO.

Heme insertion into apoproMPO occurs in the ER, as disruption of the Golgi by treating promyelocytes with brefeldin A arrests MPO biosynthesis in the proMPO stage but has no impact on correct heme incorporation (17). In both wild-type proMPO and mature MPO the posttranslationally modified heme group significantly contributes to the overall stability through covalently linking the N- and C-terminal regions of the two chains as observed by Banerjee *et al*. (40). In the absence of the Cys-158–Cys-319 disulfide bridge, heme occupancy was diminished, was reflected by the significantly reduced enzymatic activity of the respective mutants. Typically, the apoform of heme proteins (and also apoproMPO) acquires the prosthetic group via the main substrate access channel (41). As our studies have demonstrated, this access channel was structurally apart from the interface between the propeptide, the core protein, and the bridging disulfide bond (Fig. 5*A*). This finding supports the hypothesis that disruption of the Cys-158–Cys-319 disulfide bridge destabilizes the fold integrity of apoproMPO, thus compromising stable heme incorporation.

Wild-type proMPO is catalytically fully active. In recent studies it has been demonstrated that recombinant monomeric proMPO shares almost identical spectral and enzymatic features with the mature dimeric leukocyte enzyme (12, 38, 42, 43), and this similarity extends to the redox thermodynamics of the Fe(III)/Fe(II) couple (11). Furthermore, intracellular MPO and secreted proMPO from HEK cell cultures show almost identical enzymatic activities. The structure of proMPO that we reported here fully supports these findings, because it demonstrates that its core structure, including the substrate access channel and heme cavity architecture, was almost identical to that of the protomer of mature MPO. Moreover, the crystal structure clearly demonstrates that the posttranslational autocatalytic modifications of the prosthetic group (9) were already established in proMPO, suggesting that the formation of these covalent bonds must occur in the ER. The reaction requires H_2_O_2_ to generate compound I in order to oxidize the nearby carboxylate groups of Asp-260 and Glu-408 and most probably involves the formation of a carbocation followed by hydrogen abstraction from the heme methyl groups (9). A comparable mechanism has been proposed for the autocatalytic formation of the sulfonium-ion linkage that occurs in MPO but not in related peroxidases (9).

Additionally, our proMPO structure demonstrates that the formation of two covalent bonds at pyrrole ring A of proMPO by neighboring Glu-408 and Met-409 destabilized the ester but not the sulfonium bond, a phenomenon already seen in mature leukocyte MPO (8). Catalysis of chloride oxidation to antimicrobial hypochlorous acid needs the presence of an intact electron-withdrawing sulfonium-ion linkage (44–46), whereas the impact of the ester bond with Glu-408 on activity seems to be less important. Furthermore, the almost identical heme-cavity architecture of proMPO and MPO was evident by inspection of (i) the close proximal His-502–Asn-587–Arg-499 interaction and the resulting anionic character of His-502, (ii) the distal-side catalytic residues, and (iii) the geometry of the distal Ca^2+^-binding site. This clearly suggests that the active site is preassembled in proMPO.

After heme insertion the enzymatically active proMPO undergoes a series of proteolytic events. The present study suggests that the first and most important step is the elimination of the propeptide by proteolytic cleavage, a process that earlier studies suggest is mediated by a proconvertase in the Golgi (21). The overall structure of proMPO must be preserved for successful transport from the ER to the trans-Golgi, as disruption of the Cys-158–Cys-319 bond kept proMPO associated with CLN in the ER. To eliminate the propeptide, a protease needs access to the target sequence in the C-terminal region of the propeptide. The flexible nature of the propeptide, reflected by the absence of electron density for residues Ala-49–Ser-156 in the crystal structure, and its loose interaction with the core protein should facilitate access of the subtilisin-like proconvertase to its arginine-rich binding site in the propeptide. The solvent-accessible volume at the interface between the propeptide and the core protein in proMPO was significantly bigger than that between the two protomers in MPO (Fig. 7). Mutation of the target sequence in the propeptide blocks processing of proMPO and results in unstable intracellular forms (21). Our hybrid model of proMPO suggests that at least three basic amino acids of this target sequence are surface-exposed and can interact with the protease.

Simultaneously with the proteolytic event, the Cys-158–Cys-319 bridge is most likely cleaved, and a 74-kDa intermediate species is formed, thereby enabling dimerization, because both Cys-319 and the glycans at Asn-323 and Asn-483 would be accessible in the resulting 74-kDa protomers. Moreover, the elimination of the propeptide additionally exposes the hexapeptide loop for proteolytic cleavage (Figs. 1 and 4*C*). From a structural point of view, the latter proteolytic event could take place in either the monomeric or the homodimeric state. In any case, it cleaves the protomer into the L- and H-chain. The purpose of this step is unclear because the structure in mature MPO shows an almost continuous electron density between the resulting C terminus of the L-chain and the N terminus of the H-chain (8), suggesting a direct interaction between the two polypeptides. Thus the impact of this maturation step on the conformational stability and on catalytic properties should be negligible. Finally, after transport from the ER and through the complex Golgi network, the resulting mature MPO reaches its final intracellular destination in azurophilic granules.

Altogether, we solved the first crystal and solution structure of proMPO, thereby providing new insights into myeloperoxidase processing and targeting. The most interesting finding concerns the Cys-158–Cys-319 disulfide bridge between the structured but very flexible propeptide and the core protein of proMPO, which supports the proper folding of apoproMPO and heme incorporation in the ER. Heme incorporation into the pre-formed cavity is followed by autocatalytic processing and the formation of three covalent bonds between the prosthetic group and the protein. The propeptide hinders dimerization and formation of the Cys-319–Cys-319 disulfide bridge in mature homodimeric MPO. After transport to the trans-Golgi, the loose interaction between the flexible and solvent-exposed propeptide and the core protein in proMPO enables the access of a subtilisin-like proconvertase to its arginine-rich binding site in the C-terminal region of the propeptide. Finally, cleavage of the propeptide enables access of another protease to eliminate a hexapeptide loop in the resulting 74-kDa intermediate, thereby forming the L- and H-chain of the mature protomer of leukocyte MPO.

## Experimental procedures

### Materials

Highly purified dimeric leukocyte myeloperoxidase with a purity index (*A*_428_/*A*_280_) of at least 0.85 was purchased as lyophilized powder from Planta Natural Products (Vienna, Austria). Heterologous expression, purification of recombinant monomeric proMPO in CHO cells, and characterization have been described (25, 38). The purity index (*A*_428_/*A*_280_) of recombinant proMPO varied between 0.61 and 0.65. The concentration of both MPO forms was determined spectrophotometrically using ϵ_428_ = 91,000 m^−1^ cm^−1^.

Human erythroleukemia cell K562 (CCL-243) and HEK293 cells (CRL-1573) were obtained from American Type Culture Collection (Manassas, VA). The vectors pREP10 and pcDNA3.1 and the antibiotics hygromycin and G-418 sulfate were obtained from Invitrogen. [^35^S]Methionine/cysteine (Easy Tag Expre^35^S^35^S protein labeling mix, 37.0 TBq/mmol, 11 mCi/ml) was obtained from PerkinElmer Life Sciences. All tissue-culture reagents were obtained from the hybridoma facility at the University of Iowa. Antibody against calnexin was obtained from Stressgen Bioreagents (Ann Arbor, MI). The monospecific rabbit antibody against human MPO was generated in our laboratory as described previously (47), as was the rabbit antibody against human calreticulin (23), which is also available commercially from Thermo Fisher Scientific (antibody PA3-9000).

Unless specified otherwise, all other reagents were purchased from Sigma-Aldrich.

### Stably transfected cell lines

Clones of stably transfected cell lines were created and maintained, using pREP10 in K562 cells and pcDNA3.1 in HEK cells as described previously (23, 24, 30, 31, 48). PCR was used to create specific mutations in wild-type MPO cDNA for stable heterologous expression in K562 or HEK cells, which are devoid of endogenous MPO. The cDNA was sequenced prior to transfection to confirm the presence of the desired mutation and the absence of unintended mutations. Once the sequence was confirmed, stable transfectants were selected and cloned by limiting dilution.

### MPO biosynthesis by transfectants

Stably transfected cells were used to examine the biosynthesis of wild-type and mutant forms of MPO as done previously (23, 24, 30, 31, 48). In summary, cells were grown at low density in medium supplemented with 2 μg/ml hemin for 24 h prior to biosynthetic radiolabeling and then placed in methionine-free medium supplemented with hemin, dialyzed fetal bovine serum, and antibiotics for 1 h prior to pulse labeling with [^35^S]methionine/cysteine. After the indicated period of biosynthetic radiolabeling, the cells were recovered or chased by the addition of cold methionine (1000-fold excess) before solubilization for subsequent analysis. Radiolabeled cells were solubilized and used in immunoprecipitations as described previously (24, 30, 48).

Biosynthetically radiolabeled MPO-related proteins were recovered from cell lysates or culture medium by immunoprecipitation with monospecific polyclonal rabbit anti-human MPO as described previously (16, 17, 22–24, 30, 31, 48). To recover MPO-related proteins associated with CRT or CLN, we performed sequential immunoprecipitation as described previously (23, 30, 31). Cell lysates were immunoprecipitated first with antibodies against CRT or CLN under nondenaturing conditions. The CRT- or CLN-associated proteins in the recovered complex were released by heating in the presence of 2% SDS, and the solution was cooled and diluted 10-fold before proceeding with an immunoprecipitation with MPO antiserum. Radiolabeled MPO-related proteins were separated by SDS-PAGE followed by autoradiography and quantitated by direct measurement of radioactivity using a PhosphorImager (Typhoon 9410, GE Healthcare).

### Secreted proMPO

Secreted proMPO was isolated and analyzed as described previously (21). For analysis of secreted proMPO, the spent culture medium was collected from HEK cells, both wild-type and transfectants, after cells had reached ∼80% confluence in T162 culture flasks. The spent medium was clarified by centrifugation, diluted 1:1 with 10 mm Tris-HCl, pH 7.4, and incubated with SP Sepharose Fast Flow beads (GE Healthcare) that had been washed in 10 mm Tris-HCl, pH 7.4. After tumbling in diluted medium, the beads were pelleted, resuspended in Tris-buffered saline (TBS) pH 7.4, and washed three times in TBS. Bound proMPO species were eluted from beads in 1.5 m NaCl in Tris-HCl, pH 7.4, at 4 °C. The eluted supernatant was dialyzed against PBS and concentrated ∼3-fold using an Amicon Ultracel (30K). The protein concentration was determined using the Pierce BCA protein assay. The amount of MPO-related protein recovered as secreted proMPO was determined by immunoblotting. Samples were subjected to SDS-PAGE and blotting as described previously, with blots incubated with MPO antibody (1:20,000 dilution) followed by donkey anti-rabbit antibody conjugated with horseradish peroxidase (1:50,000) and processed with Pierce West Femto chemiluminescence reagent. The chemiluminescent signal was quantitated directly using a Typhoon 9410 PhosphorImager.

### Peroxidase activity

The peroxidase activity of the cell lysates as well as proMPO recovered from spent culture medium was quantitated spectrophotometrically using a modified version of a published technique (49). Cell pellets were solubilized in 0.2% (v/v) Triton X-100/PBS at a density of 1 × 10^6^ cells/20 μl and stored on ice. Peroxidase assay was performed in a water bath held at 37 °C. The reaction mixture contained 20 μl of cell lysate, 380 μl of assay buffer (1.4 mm tetramethylbenzidine, 8.8% (v/v) dimethylformamide, and 50 mm sodium acetate, pH 5.4), and 300 μm H_2_O_2_ (verified spectrophotometrically using ϵ_240 nm_ = 43.6 m^−1^ cm^−1^). The reaction was stopped after the addition of 1.7 ml of ice-cold 0.2 m acetic acid. Absorbance at 655 nm was measured spectrophotometrically. Assays of cell lysates used 5 × 10^5^ cell equivalents, whereas 10 μg of protein was used to assess the peroxidase activity of secreted mutant proMPO and 1 μg of secreted wild-type MPO.

### Differential scanning calorimetry

Differential scanning calorimetric (DSC) measurements were performed using a VP capillary DSC microcalorimeter from MicroCal with a cell volume of 137 μl. The measurements were controlled by the VP viewer program, and the instrument was equipped with an autosampler for 96-well plates. Samples were analyzed using a programmed heating scan rate of 60 °C h^−1^ over a temperature range from 20 to 100 °C, and cell pressure was ∼60 psi (4.136 bar). DSC thermograms were corrected for buffer baseline and protein concentration. The conditions were: 14.3 μm recombinant proMPO or 8.1 μm mature leukocyte MPO in PBS buffer, pH 7.4. For data analysis and conversion, MicroCal Origin software was used. Heat capacity (*C_p_*) was expressed in kcal mol^−1^ K^−1^. Data points were fitted to non-two-state equilibrium-unfolding models by the Levenberg-Marquardt nonlinear least squares method.

### Crystallization, data collection, structure determination, and refinement

Crystals of recombinant proMPO were obtained initially in the Morpheus crystallization screen (Molecular Dimensions Limited Ltd., Suffolk, United Kingdom) using the sitting-drop vapor-diffusion technique and a nanodrop-dispensing robot (Phoenix RE, Rigaku Europe, Kent, United Kingdom) and optimized to 10% (w/v) PEG 20,000, 20% PEG 550 monomethyl ether, 0.1 mm Tris-Bicine, pH 8.5, and 0.05 mm CaCl_2_ using the hanging-drop vapor-diffusion technique at 22 °C. The crystals were flash-cooled directly from the mother liquor in liquid nitrogen prior to data collection.

The data set was collected at the ESRF Synchrotron (Grenoble, France) at beamline ID29 at 100 K using a wavelength of 0.98 Å. The data frames were processed using the XDS package (50) and converted to MTZ format using AIMLESS (51). The structure was solved by molecular replacement with the program PHASER (51, 52) using atomic coordinates of human MPO (PDB accession code 1MHL) as a search model. The structure was refined using the programs REFMAC (51, 53) and Phenix Refine (54) and rebuilding was done using the program Coot (55). Coordinates were deposited in the Protein Data Bank (PDB code 5MFA). The data collection and refinement statistics are reported in Table 1. The stereochemistry and structure quality were checked using the program MolProbity (56).

Analysis of the substrate access channel in MPO and proMPO were performed using the tool CAVER 3.0 (57). Figs. 4–6 were produced using the program PyMOL (58).

### Small-angle X-ray scattering

Dimeric, mature MPO and recombinant proMPO were concentrated to 1 mg/liter and stored at 4 °C. For both systems the scattering data were collected at SIBYLS beam line 12.3.1 at the Advanced Light Source at Lawrence Berkeley National Laboratory following standard procedures (59). Both proteins were spun at 3000 rpm for 10 min and kept at 10 °C before data collection. Finally, samples were loaded into a SAXS cuvette and kept at 20 °C for 15 s. For each sample, four data sets were accumulated, namely for 0.5, 1, 2, and 4 s of radiation exposure. Data sets were background-corrected and analyzed for radiation damage. No radiation damage was observed, and background-corrected data sets accumulated after 0.5 s were chosen for further evaluation. From the scattering data we computed the pair-density distribution for MPO and proMPO.

Because we had crystal structures of proMPO and MPO in hand, we did not follow the usual approach that computes SAXS models by a reverse Monte Carlo method and tempered annealing procedures followed by matching the sphere model and the crystallographic models. We computed a centroid for each amino acid (*i.e.* potential scattering site) and attributed a particular weight to each. Next, we analytically computed the pair-density distribution for the 3D model and adapted the weight of each site until this pair-density contribution fitted to the pair-density contribution calculated from the experimental data. Initially, each site was weighted as 1. In the case of proMPO, the weights of the scattering sites of the core domain were kept constant, whereas those of the propeptide model were varied to compensate for the differences in the pair-density distributions.

Calculation of χ2, Guinier, and normalized Kratky plots were performed as described in the literature (60–62). Additionally, bead model structures for proMPO were calculated by using the software ATSAS 2.7.2 (EMBL, Hamburg, Germany).

### Mass spectrometry

The relevant protein bands were *S*-alkylated with iodoacetamide and digested in-gel with trypsin (Promega). Alternatively, the same procedure was also performed in solution. The digested samples were loaded on a BioBasic C18 column (150 × 0.32 mm, 5 μm, Thermo Fisher Scientific) using 65 mm ammonium formiate buffer as the aqueous solvent. A gradient from 5% B (B: 100% acetonitrile) to 32% B in 35 min was applied followed by a 15-min gradient from 32% B to 75% B to facilitate the elution of large peptides. The flow rate was 6 μl/min. Detection was performed with QTOF MS (Bruker maXis 4G) equipped with the standard ESI source in positive-ion DDA mode (switching to MS/MS mode for eluting peaks). MS scans were recorded (range, 150–2200 Da), and the six highest peaks were selected for fragmentation. Instrument calibration was performed using an ESI calibration mixture (Agilent Technologies). Using data analysis software from Bruker, the files were converted to mgf files, which are suitable for performing a MS/MS ion search with GPM (global proteome machine). Additionally, manual searches were made. N-terminal sequencing of proMPO by Edman degradation was performed by Dr. Bettina Sarg from the Division of Clinical Biochemistry at the Medical University in Innsbruck, Austria.

## Author contributions

C. O., W. M. N., and K. D. conceived and coordinated the study and wrote the paper. I. G. performed proMPO crystallization, data collection, structure determination, and refinement. M. P., J. S., S. H., M. S., B. S., and P. G. F. produced and characterized MPO and proMPO. R. T. was responsible for SAXS data collection and structure determination, and C. O. created the hybrid model of proMPO.

## Footnotes

4 The abbreviations used are:

MPO: myeloperoxidase
proMPO: promyeloperoxidase
CRT: calreticulin
aa: amino acid
CLN: calnexin
ER: endoplasmic reticulum
PDB: Protein Data Bank
SAXS: small-angle X-ray scattering
DSC: differential scanning calorimetry
Bicine: *N*,*N*-bis(2-hydroxyethyl)glycine.

## Note added in proof

During the preparation of the galley proofs for publication, we became aware of our unintentional oversight of a publication describing the importance of Cys-319 in production of MPO expressed in two malignant cell lines. The mechanism responsible for the faulty synthesis of MPO with mutated Cys-319 was not identified, but the phenomenon was demonstrated in the following paper: Laura, R. P., Dong, D., Reynolds, W. F., and Maki, R. A. (2016) T47D cells expressing myeloperoxidase are able to process, traffic and store the mature protein in lysosomes: Studies in T47D cells reveal a role for Cys319 in MPO biosynthesis that precedes its known role in inter-molecular disulfide bond formation. *PLoS One* **11,** 0149391.
